# Treatment outcome of tuberculosis patient of Samtse General Hospital, Bhutan

**DOI:** 10.3126/nje.v10i3.28397

**Published:** 2020-09-30

**Authors:** Thinley Dorji, Kinley Wangdi

**Affiliations:** 1 Kanglung Hospital, Trashigang, Bhutan; 2 Department of Global Health, Research School of Population Health, Australian National University, Canberra, Australia

**Keywords:** Asia, Mycobacterium Infections, Patient Outcome Assessment, Therapy, Successful

## Abstract

**Background:**

Tuberculosis (TB) is one of the major public health problems in Bhutan. Evaluation of treatment outcomes of TB and identification of the risk factors are important components for the success of National TB control program. Therefore, this study was undertaken to assess the TB treatment outcome and factors associated with it in Samtse General Hospital.

**Methods:**

This was a retrospective, cross sectional study using the TB data from Samtse General Hospital from 2008–2019. A univariate and multiple logistic regression was used to check for associations between the outcome and other independent variables.

**Results:**

The study included a total of 634 TB patients. Of this, 44.0% (279) were smear positive TB (PTB+), 36.1% (229) were extra pulmonary TB (EPTB) and 19.9% (126) were smear negative TB (PTB-). During the study period, 56.2% (356) of them completed treatment, 33.3% (211) were declared cured, 0.2% (1) had defaulted, 5.1% (32) died and 5.4% (34) had treatment failure. The mean treatment success rate (TSR) was 89.4% (567). The TSR was highest for EPTB with 96.9% (222/229), followed by PTB- at 88.1% (111/126) and lowest for PTB+ with 83.9% (234/279). Successful treatment outcome was observed in EPTB patients (AOR: 7.3; 95% CI: 2.46-21.36), patients in age 15-28 years (AOR: 3.4; 95% CI: 1.59-7.46) and 29-42 years (AOR: 9.1; 95% CI: 2.44-33.61).

**Conclusion:**

The treatment outcome of TB in Samtse General Hospital is satisfactory and at par with the national level. Since, smear positive TB and elderly patients are prone to develop poor treatment outcome, they need to be monitored and followed up adequately.

## Introduction

Tuberculosis (TB) is caused by Mycobacterium tuberculosis that primarily affects lungs (Pulmonary TB (PTB)) and other body parts known as extra-pulmonary TB (EPTB). As per the World Health Organization (WHO), 1.7 billion people are estimated to be infected with TB bacillus. Of this, 5 to 10% of them are at the risk of developing active TB during their lifetime [1]. TB caused an estimated 10 million infections in 2018 with over 1.45 million deaths [1]. The South-East Asia region accounted for highest burden of TB, attributing to 44% of the global disease burden [1].

In Bhutan, TB is a common public health problem with an incidence rate of 149 per 100,000 population [1]. The National Tuberculosis Control Program (NTCP) under the Department of Public Health is responsible for TB prevention and control in Bhutan. Piloting of short course chemotherapy for TB was initiated in 1988 followed by nationwide implementation in 1994. Bhutan started implementing Directly Observed Treatment Short-course (DOTS) and achieved 100% coverage by 1997 [2]. Since then TB treatment protocol underwent many changes. Until 2006, the TB drugs were given in loose formulations of four different drugs for intensive treatment for two months followed by fixed drug combinations for another six months. Subsequently, the TB drugs are given as fixed dose combinations with two months of isoniazid (H), rifampicin (R), pyrazinamide (Z) and ethambutol (E) in intensive phase and four months of HR in continuation phase for category 1 patients. The patients on category 2 receive two months of injection streptomycin along with three months of HRZE and five months of HRE [2]. These treatments are provided through 32 health centres which have diagnostic facilities for TB [3]. The common method of TB diagnosis is through sputum microscopy and chest X-ray. However, Gene Xpert are available in the regional referral hospitals and a few district hospitals. This is used mainly for drug susceptibility testing and not for routine diagnosis of TB. Presently, the drug resistant TB patients are referred to Mongar Regional Referral Hospital for Eastern Bhutan, Gelephu Regional Referral Hospital for central region and Gidakom Hospital for Western Region and Southern Region. These patients receive treatment as per the WHO guideline using shorter and longer regimen [4].

In Bhutan, there are no private practices and all medical services are provided by the state for free including the treatment for TB. Bhutan is pursuing the global and regional drive to eliminate TB by 2035 [4]. Therefore, the “End TB” strategy which aims to reduce the mortality due to TB by 95% and morbidity by 90% by the year 2035, has been included in the national strategic plan [5]. The TB treatment success rate (TSR) in Bhutan was 93% among drug-sensitive TB patients (new and relapse cases) in 2017 and 91% among multi-drug resistant TB (MDR-TB) in 2016 [1]. To achieve the targets set by WHO for End TB strategy, the NTCP has come up with national plan which aims to detect a minimum 90% of TB infections and maintain high TSR [4].

According to the WHO, monitoring of TB treatment outcome should be an integral part of the TB program and serve as an indicator of the performance of the NTCP [6]. It enables the program managers to identify the problems and take corrective measures and should be taken at all levels of health systems from peripherals to national [6]. Therefore, the aim of this study was to assess the treatment outcome and understand the covariates for successful treatment outcome in Samtse General Hospital.

## Methodology

### Study design and participants

This was a retrospective cross-sectional study conducted among the TB patients registered in Samtse General Hospital. All TB patients receiving treatment from 1st January 2008 to 31st December 2019 were included in this study.

### Data Collection

The data for the study was obtained from the TB treatment card and TB data-base maintained at Samtse General Hospital.

The treatment card contains information on demographic data, type of TB, treatment category, patient weight (at the time of initiation of treatment) and the outcome of the patient. These data were extracted to a standard questionnaire which was latter entered into Microsoft Excel.

### Inclusion and exclusion criteria

All TB patients who had been registered in the TB unit of Samtse General Hospital in the study period were involved in the study. However, patients with missing outcomes in the TB registers and cards were excluded from the study.

### Outcome variables

Successful treatment outcome: This group included patients who were declared cured (defined as smear or culture positive patients who become smear/culture negative in last month of treatment and at least on one occasion before) [6] and treatment completed (defined as patients who have completed the TB treatment but without laboratory confirmation of being smear/culture negative in last month of treatment or one occasion before) [6].

Unsuccessful treatment outcome: This includes patients who defaulted, those who had treatment failed, died, or were diagnosed with multi-drug resistant TB.

### Explanatory variables

Treatment category:

Category 1: The treatment is given to newly diagnosed TB patients for period of six months.

Category 2: The treatment is given to those with treatment failure, default or relapse for period of eight months with two months of injection.

Disease site:

Smear positive pulmonary TB (PTB+): At least one or more sputum smear is positive for acid fast bacilli before treatment [6].

Smear negative pulmonary TB (PTB-): The sputum is smear negative but culture positive for mycobacterium tuberculosis or at the discretion of the clinician based on the radiologic finding [6].

Extra-pulmonary tuberculosis (EPTB): Infection of organ other than the lungs [6]. The diagnosis is made based on the histo-pathological examination of the samples or fluid analysis.

Type of patient:

New: the patient has never been treated for TB or has taken TB drugs for less than one month [7].

Relapse: The patient previously received TB treatment and was declared cured/treatment completed but presenting with recurrent episode of TB [7].

Treatment after default: The patient is put on TB treatment after he/she has interrupted the anti-TB drugs for at least two consecutive months or more.

Treatment after failure: The patient whose sputum smear or culture is positive after five or more months of treatment [7].

### Ethical Clearance:

The study was approved by the Research Ethics Board of Health (Ref. No. REBH/Approval/2019/026) under the Ministry of Health, Bhutan. In order to maintain confidentiality, the name or their registration numbers were not included but were given unique identifiers.

### Sample size calculation:

A previous study among EPTB patients in Bhutan showed the overall treatment success rate of 90% [8]. Using the formula n=z2*P (1-P)/d^2^, taking 95% confidence interval and precision of 5%, the minimum sample size required was 139. However, all the TB patients registered in the Samtse General Hospital and with complete information during the study period were included.

### Data management and statistical analysis:

The data was analysed using STATA 13 (Stata Corporation, College Station, TX, USA) software. Descriptive analysis is presented in tables with frequency and percentages. Univariate and multiple logistic regression was used to check for associations between the outcome and other independent variables. P-value of <0.05 was considered as the statistically significant.

## Results

### Socio-demographic and clinical characteristic:

**Figure 1:**
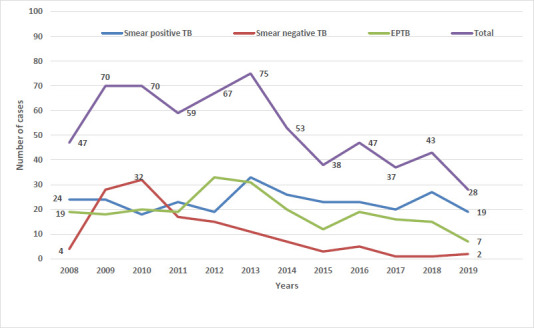
Trend of tuberculosis in Samtse General Hospital, Bhutan from 2008-2019

A total of 634 TB patients had received treatment in Samtse General Hospital in the years from 2008 to 2019 (Table 1). More than half of the patients were male (57.6%) and in the age groups of 15-42 years (61%). The mean age of the respondents were 33.4 years (SD 19.2). Of the total patients, 521 (82%) were new patients, 58 (9.2%) were transfer in, 44 (6.9%) were relapse and 8 (1.4%) were treatment default or failure. PTB+ accounted for 44% of total patients followed by EPTB at 36.1% and PTB- at 19.9%. The total number of PTB+ cases remained almost similar over the last twelve years. However, the EPTB cases increased till 2012 and decreased thereafter. Similarly, the number of PTB-cases increased from 2008 till 2010 after which there was a decline of cases (Fig.1). During the study period, 29 cases of MDR TB were detected.

### Treatment outcome of TB

Of the total patients, 356 (56.2%) had completed treatment, 211 (33.3%) were declared cured and 34 (5.4%) had treatment failure. The case fatality rate from TB during the study period was 5.1% (32 patients). Of these, 78% (25) were above the age of 56 years (mean age 64.72 ± 13.17). However, there were no TB related deaths in 2018 and 2019.The proportion of cured patients increased over the years while that of treatment completed decreased over the years. (Table 2)

### Treatment success rate (TSR)

**Fig 2:**
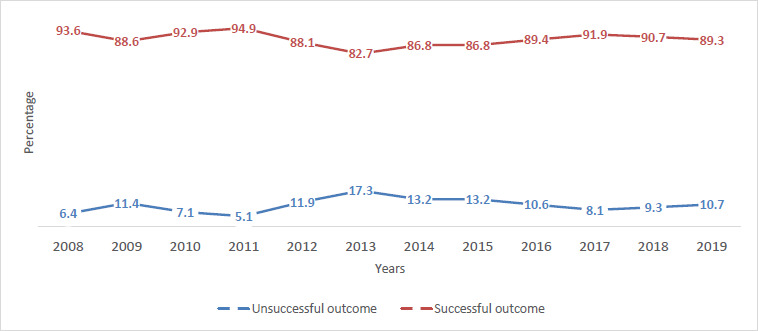
Trend of treatment success rate of TB in Samtse General Hospital, 2008-2019

The mean TSR in the last 12 years was 89.4% (567/634). The TSR was highest for EPTB (96.9%), followed by PTB- (88.1%) and lowest for PTB+ (83.9%). The TSR for TB was highest in 2011 at 94.9% which decreased to as low as 82.7% in 2013. However, TSR reached 89.3% in 2019 (Fig. 2).

Determinants of TB treatment outcome

The determinants of treatment outcome were analysed using bivariate analysis. There were significant differences among age-groups, types of TB and category of TB between successful and unsuccessful treatment outcome. Using multiple logistic regression and adjusting for sex, age group, weight of the patient, treatment category and type of patients, factors that were still statistically significant with successful outcome were EPTB (AOR:7.3; 95% CI: 2.46-21.36 p-value-<0.001), age group 15-28 years (AOR:3.4; 95% CI:1.59-7.46, p-value-0.001) and age 29-42 years (AOR: 9.1; 95% CI: 2.44-33.61, p-value-0.001) (Table 3). However, both the variables had relatively wide confidence interval. The PTB- were more likely to have successful treatment outcome compared to PTB+ though it was not statistically significant (AOR:2; 95% CI:0.84-4.63, p-value).

## Discussion

This study involved retrospective analysis of the TB data from Samtse General Hospital for last twelve years from 2008 to 2019. It was conducted in one of the high TB burden districts of Bhutan and showed mean TSR of 89.3%. The correlates for successful TB treatment outcome were patients with extra-pulmonary TB, patients in age group of 15-28 years and 29-42 years.

### TB treatment success rate

The TSR of Samtse General Hospital is at par with the National TSR of Bhutan (93%) and the global TSR (85%) [9]. This is in agreement to studies from countries of Nepal (91%), Bangladesh (94%), Sri Lanka (85%), Pakistan (93%) [9] and Ethiopia (92.5%) [10]. The TSR in our study is higher than the cumulative TSR of India (81%) [10]. However, the TSR in India varies from 51.5% in Chhattisgarh, 84.6% among disadvantaged population in New Delhi to 86% in Chennai [11-13]. Similarly a nationwide study on EPTB in Bhutan showed TSR of 90% [8]. The lowest TSR for Samtse General Hospital was observed in 2013 which might be due to increased detection of drug resistant TB [14] after the introduction of liquid culture in Bhutan. The TSR of Samtse General Hospital increased from 83% in 2013 to 89% in 2019. One reason for the increasing trend of TSR in this study could be as a result of good performance of DOTS. Similar findings have been reported in other countries [15].

### Risk factors of unsuccessful TB treatment outcome

Patients with EPTB have better treatment outcome than PTB+ which was similar to other studies [11, 16]. This could be due to the fact that EPTB occurs in young age groups [17]. Moreover, the treatment outcome for EPTB is based on the clinical remission and completion of treatment rather than on bacteriological test as in case of PTB+. In contrast, a study in Ethiopia observed PTB+ had better treatment outcome compared to EPTB and PTB- [15, 18]. This was thought to be due to high prevalence of HIV among PTB- patients.

There is an inverse relationship between age and the treatment outcome. Old age is associated with poor treatment outcome. This is consistent with other studies where TB patients with younger age groups are associated with better outcome compared to older age groups [11, 16, 19-21]. This could be due to presence of various co-morbid illness, weakening health and immunity at old age. However, a study in Denmark showed that age was not an associated factor for TB outcome [22]. This could be due to difference in the definition of the unsuccessful outcome since the above-mentioned study excluded TB death from unsuccessful outcome.

Studies have shown that male patients are significantly associated with poor outcome [10, 16, 18, 20, 22, 23], which could be due to high default rates among male patients. Further, males are associated with risky behaviour including smoking and drinking alcohol. However, gender was not important determinant in our study as in Spain [19].

The TB associated death in this study was 5.1%. This is in line with other studies from Eastern Ethiopia (3.9%) [10] and India (6%) [24]. However, the TB death in this study is higher than a similar study conducted in Phuntsholing Hospital located in Southern Bhutan, where mortality rate was 2% [23]. The differences could be explained by the socio-economic status as Phuntsholing General Hospital caters to the city people. The treatment failure rate in this study (5.4%) was similar to India (6.3%) [11] and higher than Eastern Ethiopia (1.2%) [10]. High bacillary load, diabetes, alcohol consumers, HIV positive, smear positive at two months of treatment and weight loss are associated with treatment failure [25, 26]. However, these variables were not recorded as part of the surveillance system in our data.

## Limitation of the study

This study has several limitations that has to be considered when interpreting the findings of this study. Firstly, this is a retrospective study, so information on educational level, occupation, income and risk behaviours like alcohol and smoking of the patient which could inadvertently affect the outcome were missing in this study. Secondly, there was no data on HIV co-infection TB data due to confidentiality issue. Given its retrospective study, it cannot establish causal relations, but rather can only generate hypotheses that could be evaluated by future prospective randomized trials.

## Conclusion

The TSR from this study was 89.3%. This is comparable to the national and global TSR of >90%. Patients with extra-pulmonary TB and younger patients were individual positive predictors of successful TB outcome. Therefore, smear-positive TB and older patients need additional care to further increase TSR.

### Future scope of the study

Although this study showed good TB treatment outcome in Samtse Hospital, the results cannot be generalized. Therefore, a larger study including many TB treatment centres could give an accurate result. Other factors which affect the treatment outcome could not be accessed in this study due to limited data in the treatment card. Inclusion of pertinent data in the TB treatment card would help in the identification of factors responsible for the outcome.

### What is already known on this topic?

- TB is a major health problem in Bhutan.
- Proportion of childhood TB in Bhutan was 14% and TSR was 93%.
- Proportion of EPTB in Bhutan varied from 30-40% during 2001-2010. Tuberculous lymphadenitis and pleural effusion were the two most common types of EPTB. The TSR of EPTB was 90%.
- Annual rate of TB infection among children aged 6-8 years in Bhutan was 0.2-0.7% [27].

### What this study adds:

This study presents TSR (Treatment Success rate) in Samtse district in Bhutan. The individual level factors associated with TSR.

## Figures and Tables

**Table 1:** Socio-demographic and clinical characteristic of the tuberculosis patients in Samtse General Hospital, 2008-2019

Characteristics		Number (%)
**Sex**		
	Male	365 (57.6)
	Female	269 (42.4)
**Age (years)**		
	<=14	64 (10.1)
	15-28	290 (45.7)
	29-42	101 (15.9)
	43-56	73 (11.5)
	>=56	106 (16.7)
**Baseline Weight (kg)**		
	< 30	41 (8.4)
	30-59	388 (79.5)
	60-90	59 (12.1)
**Types of TB**		
	Smear positive TB	279 (44.0)
	Smear negative TB	126 (19.9)
	Extra pulmonary TB	229 (36.1)
**Treatment category**		
	Category 1	579 (91.3)
	Category 2	55 (8.7)
**Type of patient**		
	New patient	521 (82.2)
	Relapse	44 (6.9)
	Treatment after default	2 (0.3)
	Treatment after failure	7 (1.1)
	Transfer in	58 (9.2)
	Others	2 (0.3)
**Treatment outcome**		
	Cured	211 (33.3)
	Completed	356 (56.2)
	Treatment failure	34 (5.4)
	Defaulted	1 (0.2)
	Died	32 (5.1)

**Table 2:** Treatment outcome for TB in Samtse General Hospital, 2008-2019

	2008	2009	2010	2011	2012	2013	2014	2015	2016	2017	2018	2019	Total
**Cured**	19 (40.4%)	16 (22.9%)	12 (17.1%)	20 (33.9%)	13 (19.4%)	20 (26.7%)	20 (37.7%)	18 (47.4%)	19 (40.4%)	17 (46%)	20 (46.5%)	17 (60.7%)	211 (33.3%)
**TC**	25 (53.2%)	46 (65.7%)	53 (75.7%)	36 (61%)	46 (68.7%)	42 (56%)	26 (49.1%)	15 (39.5%)	23 (48.9%)	17 (46%)	19 (44.2%)	8 (28.6%)	356 (56.2%)
**TF**	1 (2.1%)	0	0	0	3 (4.5%)	10 (13.3%)	5 (9.4%)	4 (10.5%)	3 (6.4%)	1 (2.7%)	4 (9.3%)	3 (10.7%)	34 (5.4%)
**Defaulted**	0	0	0	0	1 (1.5%)	0	0	0	0	0	0	0	1 (0.2%)
**Died**	2 (4.3%)	8 (11.4%)	5 (7.1%)	3 (5.1%)	4 (6%)	3 (4%)	2 (3.8%)	1 (2.6%)	2 (4.3%)	2 (5.4%)	0	0	32 (5.1%)
**Total**	47	70	70	59	67	75	53	38	47	37	43	28	634

**Table 3:** Factors associated with treatment outcome in Samtse General Hospital, 2008-2019

Characteristic	Treatment success		COR (95% CI)	p-value	AOR (95% CI)	p-value
	Unsuccessful	Successful				
**Sex**						
Male	41 (61.2%)	324 (57.1%)	1		1	
Female	26 (38.8%)	243 (42.9%)	1.2 (0.7-1.99)	0.53	0.8 (0.4-1.5)	0.445
**Age group (years)**						
<=14	2 (3%)	62 (10.9%)	10.6 (2.43-46.28)	0.002	2.9 (0.5-17.24)	0.233
15-28	25 (37.3%)	265 (46.7%)	3.6 (1.99-6.6)	<0.0001	3.4 (1.59-7.46)	0.001
29-42	3 (4.5%)	98 (17.3%)	11.2 (3.27-38.16)	<0.0001	9.1 (2.44-33.61)	0.001
43-56	10 (14.9%)	63 (11.1%)	2.2 (0.97-4.78)	0.059	2.2 (0.8-6.05)	0.129
>56	27 (40.3%)	79 (13.9%)	1		1	
Weight (kg)**						
<30 kg	2 (3.9%)	39 (8.9%)	Ref		1	
30-59 kg	47 (92.2%)	341 (78%)	0.4 (0.09-1.59)	0.18	0.6 (0.1-3.03)	0.501
>59 kg	2 (3.9%)	57 (13%)	1.5 (0.2-10.82)	0.71	1.7 (0.19-15.9)	0.628
**Types of TB**						
Smear positive TB	45 (67.2%)	234 (41.3%)	1		1	
Smear negative TB	15 (22.4%)	111 (19.6%)	1.4 (0.76-2.66)	0.27	2 (0.84-4.63)	0.119
EPTB	7 (10.4%)	222 (39.1%)	6.1 (2.69-13.81)	<.0001	7.3 (2.46-21.36)	<0.001
**Treatment category**						
category 1	55 (82.1%)	524 (92.4%)	2.7 (1.32-5.34)	0.006	1.8 (0.77-4.35)	0.171
category 2	12 (17.9%)	43 (7.6%)	1		1	

**weight available for 488 patients, COR- Crude odds ratio, AOR- Adjusted odds ratio, EPTB-extra pulmonary TB

## References

[1] World Health Organization (WHO). Global Tuberculosis Report 2019 [cited 2020 July 20] Available from: URL: https://apps.who.int/iris/bitstream/handle/10665/329368/9789241565714-eng.pdf?ua=1

[2] Ministry of Health. National guidelines for management of tuberculosis 2016 [cited 2020 Jan 15] Available from: URL: http://www.moh.gov.bt/wp-content/uploads/afd-files/2018/09/National-Guidelines-for-Management-of-TB-Sixth-Edition-2016.pdf

[3] Ministry of Health. Annual Health Bulletin 2020 [cited 2020 July 09] Available from: URL: http://www.moh.gov.bt/wp-content/uploads/ict-files/2017/06/health-bulletin-Website_Final.pdf

[4] Ministry of Health. National guideline for the management of drug resistant tuberculosis 2017 [cited 2020 Jan 20] Available from: URL: http://www.moh.gov.bt/wp-content/uploads/afd-files/2018/09/National-Guideline-For-The-Managment-of-Drug-Resistant-TB-2017.pdf

[5] World Health Organization (WHO). The end TB strategy 2015 [cited 2020 Feb 20] Available from: URL: https://www.who.int/tb/End_TB_brochure.pdf?ua=1

[6] World Health Organization (WHO). Treatment of tuberculosis Guidelines 2010 [cited 2020 July 10] Available from: URL: https://apps.who.int/iris/bitstream/handle/10665/44165/9789241547833_eng.pdf?sequence=1 23741786

[7] World Health Organization (WHO). Definitions and reporting framework for tuberculosis 2013 revision. [cited 2020 June 15] Available from: URL: https://apps.who.int/iris/bitstream/handle/10665/79199/9789241505345_eng.pdf?sequence=1

[8] Jamtsho T Harries AD Malhotra S The burden and treatment outcomes of extra-pulmonary tuberculosis in Bhutan. Public health action. 2013;3(1):38-42.10.5588/pha.12.0085. 10.5588/pha.12.0085 PMid: PMCid: 26392994 PMC4463083

[9] World Bank. Tuberculosis treatment success rate (% of new cases) 2019 [updated 14/03/2020] Available from: URL: https://data.worldbank.org/indicator/SH.TBS.CURE.ZS

[10] Tola A Minshore KM Ayele Y Mekuria AN Tuberculosis treatment outcomes and associated factors among TB patients attending public hospitals in Harar town, Eastern Ethiopia: A five-year retrospective study. Tuberculosis research and treatment. 2019; 10.1155/2019/1503219 PMid: PMCid: 31057963 PMC6463571

[11] Jackson C Stagg H Doshi A Tuberculosis treatment outcomes among disadvantaged patients in India. Public Health Action. 2017;7(2):134-40. 10.5588/pha.16.0107 PMid: PMCid: 28695087 PMC5493095

[12] Ramachandran G Kupparam HKA Vedhachalam C Factors influencing tuberculosis treatment outcome in adult patients treated with thrice-weekly regimens in India. Antimicrob Agents Chemother. 2017;61(5): e02464-16. 10.1128/AAC.02464-16 PMid: PMCid: 28242663 PMC5404592

[13] Laux TS Patil S Predictors of tuberculosis treatment outcomes among a retrospective cohort in rural, Central India. Journal of Clinical Tuberculosis and Other Mycobacterial Diseases. 2018;12 (0):41-47. 10.1016/j.jctube.2018.06.005 PMid: PMCid: 31720398 PMC6830133

[14] Dorji T. Epidemiology of Drug Resistant Tuberculosis in Samtse General Hospital, Bhutan: A Retrospective Study. SAARC Journal of Tuberculosis, Lung Diseases and HIV/AIDS. 2019;17(1):41-46. 10.3126/saarctb.v17i1.2502_7

[15] Gebrezgabiher G Romha G Ejeta E Asebe G Zemene E Ameni G Treatment Outcome of Tuberculosis Patients under Directly Observed Treatment Short Course and Factors Affecting Outcome in Southern Ethiopia: A Five-Year Retrospective Study. PLoS One. 2016;11(2): e0150560. 10.1371/journal.pone.0150560 PMid: PMCid: 26918458 PMC4769218

[16] Ditah IC Reacher M Palmer C Monitoring tuberculosis treatment outcome: analysis of national surveillance data from a clinical perspective. Thorax. 2008;63(5):440-46. 10.1136/thx.2006.073916 PMid: 17615085

[17] Sreeramareddy CT Panduru KV Verma SC Joshi HS Bates MN Comparison of pulmonary and extrapulmonary tuberculosis in Nepal-a hospital-based retrospective study. BMC Infect Dis. 2008;8(1):8. 10.1186/1471-2334-8-8 PMid: PMCid: 18218115 PMC2245948

[18] Ejeta E Beyene G Balay G Bonsa Z Abebe G Factors associated with unsuccessful treatment outcome in tuberculosis patients among refugees and their surrounding communities in Gambella Regional State, Ethiopia. PloS one. 2018;13(10):e0205468. 10.1371/journal.pone.0205468 PMid: PMCid: 30335777 PMC6193657

[19] Caylà JA Caminero JA Rey R Lara N Valles X Galdós-Tangüis H Current status of treatment completion and fatality among tuberculosis patients in Spain. Int J Tuberc Lung Dis. 2004;8(4):458-64. PMid: 15141739

[20] Falzon D Le Strat Y Belghiti F Infuso A Exploring the determinants of treatment success for tuberculosis cases in Europe. Int J Tuberc Lung Dis. 2005;9(11):1224-29. PMid: 16333929

[21] Wen Y Zhang Z Li X Treatment outcomes and factors affecting unsuccessful outcome among new pulmonary smear positive and negative tuberculosis patients in Anqing, China: a retrospective study. BMC infectious diseases. 2018;18(1):104. 10.1186/s12879-018-3019-7 PMid: PMCid: 29506480 PMC5836329

[22] Holden IK Lillebaek T Seersholm N Andersen PH Wejse C Johansen IS Predictors for pulmonary tuberculosis treatment outcome in Denmark 2009–2014. Sci Rep. 2019;9(1):1-8. 10.1038/s41598-019-49439-9 PMid: PMCid: 31506499 PMC6736960

[23] Wangdi K Gurung MR The epidemiology of tuberculosis in Phuentsholing General Hospital: a six-year retrospective study. BMC Res Notes. 2012; 5:311.10.1186/1756-0500-5-311. 10.1186/1756-0500-5-311 PMid: PMCid: 22715941 PMC3517362

[24] Jonnalagada S Harries AD Zachariah R Satyanarayana S Tetali S Keshav Chander G The timing of death in patients with tuberculosis who die during anti-tuberculosis treatment in Andhra Pradesh, South India. BMC Public Health. 2011 Dec 13;11:921. 10.1186/1471-2458-11-921 PMid: PMCid: 22166132 PMC3254139

[25] Khoubfekr H Khanjani N Jahani Y Moosazadeh M Factors Associated with Treatment Failure among Smear Positive TB Patients in Khorasan-e-Razavi and Sistan-Baluchistan Provinces, Iran. Journal of Microbiology & Infectious Diseases. 2016;6(4). 10.5799/jmid.328927

[26] Diallo A Dahourou DL Tassembedo S Sawadogo R Meda N Factors associated with tuberculosis treatment failure in the Central East Health region of Burkina Faso. Pan African Medical Journal. 2018;30(1). 10.11604/pamj.2018.30.293.15074 PMid: PMCid: 30637077 PMC6320454

[27] Wangchuk LZ Chadha VK Annual risk of tuberculous infection among schoolchildren in Bhutan. Int J Tuberc Lung Dis. 2013;17(4):468-72. 10.5588/ijtld.12.0668 PMid: 23485380

